# Digital remote monitoring for screening and early detection of urinary tract infections

**DOI:** 10.1038/s41746-023-00995-5

**Published:** 2024-01-13

**Authors:** Alexander Capstick, Francesca Palermo, Kimberley Zakka, Nan Fletcher-Lloyd, Chloe Walsh, Tianyu Cui, Samaneh Kouchaki, Raphaella Jackson, Martin Tran, Michael Crone, Kirsten Jensen, Paul Freemont, Ravi Vaidyanathan, Magdalena Kolanko, Jessica True, Sarah Daniels, David Wingfield, CR&T Group, Ramin Nilforooshan, Payam Barnaghi

**Affiliations:** 1 Imperial College London, London, UK; 2 UK Dementia Research Institute, Care Research and Technology Centre, London, UK; 3 University College London, London, UK; 4 Great Ormond Street Hospital NHS Foundation Trust, London, UK; 5 University of Surrey, London, UK; 6 Surrey and Borders Partnership NHS Foundation Trust, Leatherhead, UK

**Keywords:** Preventive medicine, Predictive markers, Computer science, Applied mathematics

## Abstract

Urinary Tract Infections (UTIs) are one of the most prevalent bacterial infections in older adults and a significant contributor to unplanned hospital admissions in People Living with Dementia (PLWD), with early detection being crucial due to the predicament of reporting symptoms and limited help-seeking behaviour. The most common diagnostic tool is urine sample analysis, which can be time-consuming and is only employed where UTI clinical suspicion exists. In this method development and proof-of-concept study, participants living with dementia were monitored via low-cost devices in the home that passively measure activity, sleep, and nocturnal physiology. Using 27828 person-days of remote monitoring data (from 117 participants), we engineered features representing symptoms used for diagnosing a UTI. We then evaluate explainable machine learning techniques in passively calculating UTI risk and perform stratification on scores to support clinical translation and allow control over the balance between alert rate and sensitivity and specificity. The proposed UTI algorithm achieves a sensitivity of 65.3% (95% Confidence Interval (CI) = 64.3–66.2) and specificity of 70.9% (68.6–73.1) when predicting UTIs on unseen participants and after risk stratification, a sensitivity of 74.7% (67.9–81.5) and specificity of 87.9% (85.0–90.9). In addition, feature importance methods reveal that the largest contributions to the predictions were bathroom visit statistics, night-time respiratory rate, and the number of previous UTI events, aligning with the literature. Our machine learning method alerts clinicians of UTI risk in subjects, enabling earlier detection and enhanced screening when considering treatment.

## Introduction

Urinary Tract Infections (UTIs) are one of the most common bacterial infections in older adults, constituting around 25% of all infections^1–5^. Clinical presentation ranges from self-limited illness to severe sepsis. UTIs account for ~9–31% of cases of severe sepsis which itself has an estimated mortality of 20–40%^4,6–9^. To differentiate between asymptomatic bacteriuria and UTIs, clinicians rely on positive findings of bacteriuria and genitourinary symptoms. Diagnosis is further complicated by the presence of cognitive impairment or dementia since People Living with Dementia (PLWD) may find it challenging to report their symptoms, and this could result in further complications^10–12^. As a result, acute infections might not be diagnosed until symptoms require hospitalisation^13^. In the United Kingdom, over 20% of hospital beds are occupied by PLWD, with 9% of these attributed to UTIs^14–17^.

Currently, a urine sample test and acute changes in baseline cognition are used to diagnose UTIs in PLWD^18^. However, samples can be difficult to obtain due to urinary incontinence, cognitive impairment, sample contamination or previous use of antibiotics^19^ and are taken on suspicion of an infection, which may be delayed. Additionally, although they can be used as rapid detectors, dipstick tests have a high false positive rate for older adults and require action from the PLWD or their carer which limits their effectiveness for diagnosis^3,20^. Highlighting UTI risk by identifying early symptoms would allow for prompt diagnosis, improved health outcomes and effective allocation of healthcare resources.

Machine Learning (ML) offers opportunities for clinical diagnosis and decision-support and recent advances show promise for development of advanced predictive models that incorporate patient data to improve diagnostic performance. For UTI detection in PLWD, ML can improve diagnostic performance and timeliness. Several investigations have been conducted for UTI risk prediction on younger adult populations^21,22^, which do not generalise to older adults. Existing methods developed for older adults also rely on typical symptoms as predictor variables, precluding their use in community-dwelling patients with dementia with atypical clinical manifestations and who may struggle expressing symptoms^23^. In parallel, low-cost monitoring devices have been developed to offer complementary solutions to the typical diagnostic criteria^24,25^. Rantz et al.^26^ use activity data collected from in-home Passive Infra-Red (PIR) sensors to detect UTIs in older adults. However, their work is limited to 37 participants and does not utilise ML techniques. The study is also limited to the use of activity data and does not utilise physiological measurements. Work by Enshaeifar et al.^17^ employed an unsupervised approach to predict UTIs based on in-home sensors and physiological measurements, however their work showed insufficient diagnostic performance and required the participant to record their own physiology measurements twice a day.

**Fig. 1:**
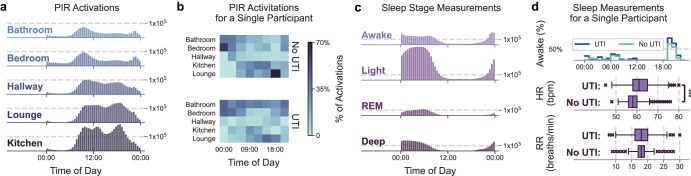
The dataset. **a** All PIR activations measured and the corresponding time of day. **b** The average proportions of PIR activations in a time period for days containing a verified positive and negative UTI label for a selected participant. The difference in Bathroom activity is of note, showing a significant increase in use for those days corresponding to a positive UTI. **c** All sleep state measurements within the dataset and the corresponding time of day. **d** The first of the graphs show the percentage of a given time window a single participant spent in bed and awake; the second and third shows the difference in heart rate and respiratory rate distributions for days labelled as positive and negative UTI for a single participant. The elements of the boxplots correspond to: center line, median; box limits, upper and lower quartiles; whiskers, 1.5x interquartile range; points, outliers. Here HR corresponds to heart rate and RR corresponds to respiratory rate.

This study presents a machine learning application to identifying the risk of UTI events in PLWD by analysing symptom-targeted features, engineered from continuous in-home activity and physiology data collected by low-cost and passive sensors (Fig. 1 presents an overview). Then, through optimisation and consultations with clinicians, we determine thresholds for the stratification of the risk scores to improve the algorithm’s clinical applicability. The proposed approach has been evaluated in an observational clinical study consisting of 117 participants living with dementia within their own homes. We have worked closely with healthcare professionals to implement a reliable and non-intrusive UTI risk model. Our work will (1) aid clinicians in the early diagnosis of UTIs, and (2) enable a better understanding of in-home behaviour at the point of clinical decision-making. The use of high-resolution in-home observation and measurement data in conjunction with machine learning methods result in timely interventions that can have a significant impact on reducing preventable and unplanned hospital admissions in dementia patients. Such a tool allows for precise collection of urine samples for culture analysis, improved clinical outcomes, a reduction in the burden on healthcare services, and decreased antibiotic overuse and misuse in PLWD by reducing UTI detection time and providing practitioners with more complete pictures of their patients.

## Results

### Model performance

We examined Logistic Regression (LR), Extreme Gradient Boosting Decision Trees (XGBoost)^27^, Multilayer Perceptron (MLP), Self-Attention^28^, Random Forest (RF)^29^, and Naive Bayes (NB) in their effectiveness to predict UTI events and found the best-performing classification model was LR with L2 regularisation, acting over 3 consecutive days of data. Table 1 presents this model performance on the different data splits. Results from the other models are included in Supplementary Information Section 10. Analysis of model reliability and calibration can be seen in Supplementary Information Section 11.

**Table 1:** Mean (95% CI) % of sensitivity, specificity, and area under the precision-recall curve of the UTI prediction model on the different data splits with 10 bootstrap repeats.

Before risk Stratification				
		Sensitivity	Specificity	AUC Precision-Recall
Date	Validation	67.3 (65.9–68.6)	69.1 (66.9–71.4)	67.7 (66.4–69.0)
Date	Test	54.5 (52.7–56.4)	73.0 (71.2–74.8)	54.4 (53.4–55.4)
Date-ID	Validation	87.7 (85.2–90.2)	66.0 (64.4–67.7)	78.3 (76.8–79.7)
Date-ID	Test	65.2 (64.3–66.2)	70.9 (68.6–73.1)	63.5 (61.8–65.2)

After Risk Stratification				
		Sensitivity	Specificity	Precision
Date	Validation	86.6 (80.9–92.3)	94.5 (91.7–97.3)	87.3 (82.2–92.4)
Date	Test	69.0 (64.4–73.5)	94.1 (92.0–96.2)	81.9 (75.5–88.2)
Date-ID	Validation	98.3 (95.5–101.1)	90.0 (85.5–94.5)	81.7 (74.4–89.1)
Date-ID	Test	74.7 (67.9–81.5)	87.9 (85.0–90.9)	77.0 (71.9–82.1)

These results are reported before and after risk stratification is performed.

### Risk stratification

**Fig. 2:**
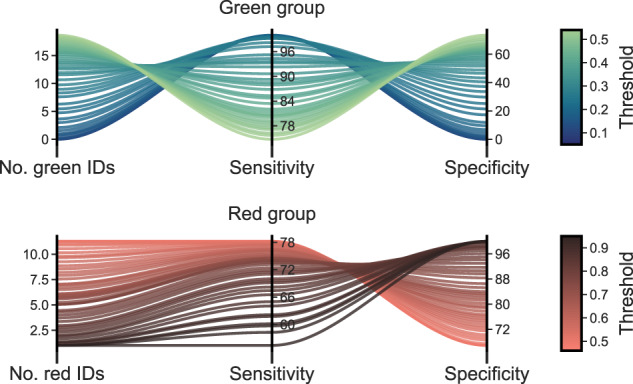
Sensitivity and specificity with different thresholds. The variations in sensitivity and specificity that can be achieved on the validation set by changing the thresholds for defining Green and Red groups. The line colours represent different threshold values. Sensitivity and specificity are calculated on the data in the validation set corresponding to Red and Green alerts. Here, to calculate the metrics on the Green group, the Red threshold was set at >50%, and when calculating the metrics on the Red group, the Green threshold was set at ≤50%. This figure shows the average results from the “Date Split" and “Date-ID Split".

To improve flexibility of the model to varying clinical settings, we calculate stratified risk scores as discussed in Section: Stratification of Risk Scores for Clinical Reporting. Figure 2 shows the sensitivity and specificity that can be achieved on the validation set by stratifying the results. By varying the stratification thresholds, sensitivity and specificity can be balanced with the number of people given Green and Red alerts. In Supplementary Information Section 12.1, we present the performance variations when jointly changing the Red and Green thresholds. Here, we select thresholds $[0 \% ,30 \% ],\left(30 \% ,80 \% \right]$, and $\left(80 \% ,100 \% \right]$ (following interval notation) for groups Green, Amber, and Red respectively. Table 1 shows the results of grouping the risk predictions on the Red and Green groups.

### Early detection

**Fig. 3:**
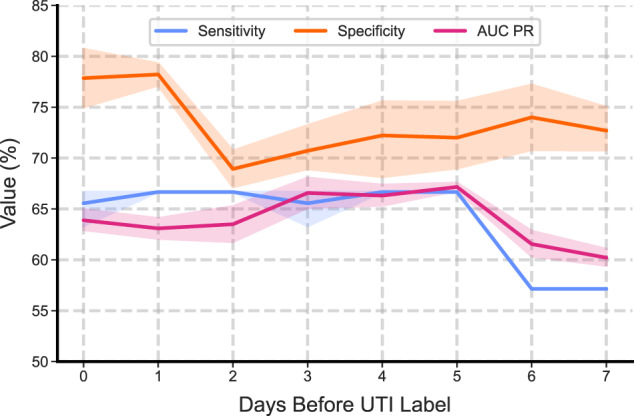
Performance at days prior to UTI label. The performance of the model when tasked with analysing the risk of a UTI from the test set, at different numbers of days prior to the verified recorded date. The error bands represent the 95% confidence interval (1000 bootstrap samples) of the mean of the values from the 10 bootstrap repeats.

We evaluated the model’s utility in correctly estimating the risk of UTIs prior to the recorded clinical urine tests. Figure 3 demonstrates specificity, sensitivity and the area under precision-recall curve for days prior to the recorded UTI events. This shows that 2 days prior to a sample test, our model achieved a sensitivity of 64.4 (95% CI = 61.1–67.8), specificity of 68.9 (95% CI = 66.8–71.0), and area under the precision-recall curve of 64.5 (95% CI = 63.0–66.0), and 4 days prior, a sensitivity of 64.4 (95% CI = 61.1–67.8), specificity of 71.9 (95% CI = 67.9–75.8), and area under the precision-recall curve of 65.4 (95% CI = 60.8–70.0).

### Feature importance

**Fig. 4:**
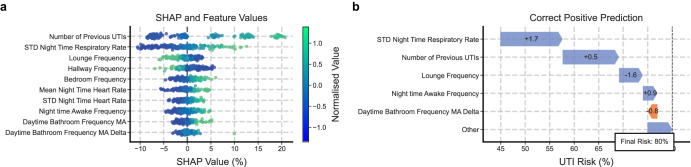
SHAP values. **a** The feature importance for the top 10 most important features, as calculated by SHAP on the test set, and their corresponding feature values. The colour represents the normalised feature value, whilst the position in the *x*-axis represents the contribution that value made to the prediction. “MA" refers to the moving average, whilst “Delta" refers to the percentage change in the value from the previous day. These values are calculated on the test set of the “Date-ID Split". **b** The breakdown of a single prediction shows how each feature contributed to a correct prediction of a positive UTI. Here, the values on the arrows correspond to the normalised feature value in units of standard deviations away from the mean.

The most important features influencing predictions were identified using SHapley Additive exPlanations (SHAP)^30^, a method for producing explainable predictions and calculating contributions from individual features to risk scores. The results of this, on the test set, can be seen in Fig. 4a and reveal that the number of previous confirmed UTI events, the standard deviation of the nocturnal respiratory rate, the nocturnal average heart rate, and the number of nocturnal awake states were positively correlated with a higher risk score. We can also breakdown single predictions to understand contributions to a risk score, as show in Fig. 4b. Further examples can be seen in Supplementary Information Section 13.

### Frequency of generated alerts

To understand the requirements of our model in a clinical setting, we calculated the risk groups of each day of data between 2022/10/05 and 2022/12/01, for each of the PLWD in our dataset. We find that on average, each of the PLWD will receive 0.25 Green alerts, 0.69 Amber alerts and 0.06 Red alerts each day. In Supplementary Information Section 14, we visualise how this risk score varies over time and in Supplementary Information Section 15, we present the model performance on subsets of the sensors. Additionally, in Supplementary Information Section 16, we compare the performance between those with recurrent and non-recurrent UTIs and in Supplementary Information Section 17, we compare the results between Male and Female participants.

## Discussion

The 2020 report of the Lancet Commission on dementia prevention, treatment, and care emphasises the significance of individualised interventions to address complex medical problems in dementia, which result in unnecessary hospital admissions, accelerated functional decline, and decreased quality of life^31^. An area of priority development is infection prevention and timely detection and treatment^32^. By conducting preliminary experiments into early identification of possible UTIs in remote healthcare settings, we hope to contribute to directly addressing this priority by investigating more individualised, predictive, and preventative healthcare.

We present a machine learning pipeline for continuous UTI risk screening via analysis of passively collected in-home activity and physiology data. We considered several models and found that LR acting over 3 days attained the top performance (sensitivity of 65.2% (95% CI = 64.3–66.2) and 54.5% (95% CI = 52.7–56.4), and specificity of 70.9% (95% CI = 68.6–73.1) and 73.0% (95% CI = 71.2–74.8) on “Date-ID Split" and “Date Split" respectively). The performance was higher on “Date-ID Split" than “Date Split", which we hypothesise is due to some PLWD who have opposing labels in the training and testing data. In this case, in “Date Split", the model might over-fit to the training data from a PLWD. However, in “Date-ID Split" all data seen by the model during testing is from participants not appearing in training. The ratios of positive to negatives in the test sets of the “Date Split" and the “Date-ID Split" are 0.31 and 0.32 respectively and 0.47 and 0.47 for the training set of the “Date Split" and the “Date-ID Split" respectively.

Through stratification, risk scores were transformed into more accessible groups, allowing for the flexible management of actionable alerts within a time period. Following this, the performance on the Green and Red groups were significantly improved, achieving a sensitivity of 74.7% (95% CI = 67.9–81.5) and 69.0% (95% CI = 64.4–73.5), and specificity of 87.9% (95% CI = 85.0–90.9) and 94.1% (95% CI = 92.0–96.2) on the “Date-ID Split" and “Date Split" respectively.

SHAP analysis then highlighted the features most strongly predictive of the risk score. Our analysis shows that an increase in the number of previously confirmed UTI events was associated with a positive UTI prediction, agreeing with the literature^33^. We also highlighted the frequency of the lounge and hallway activations as negatively correlated with risk score, whilst the bedroom frequency was positively correlated. We postulate that this results from participants spending more time in bed due to interrupted sleep, or due to the effects of comorbidities. Third, increases in the standard deviation of the night time respiratory rate and the night time average heart rate were correlated with a higher risk of a UTI. Nocturnal respiratory rate has been linked to stress, reflects physiologic and pathophysiologic determinants, and has been suggested as a biomarker for impending hospitalisation^34–38^. Increased nocturnal awake occurrences were associated with a higher UTI risk, suggesting PLWD with UTIs were having more disturbed nights of sleep; in agreement with the literature^39^. This could additionally explain why increased standard deviation of the night time respiratory rate and the night time average heart rate were correlated with a higher risk of a UTI. Considering the clinical manifestations of UTIs in older adults, our feature importance results agree with the current understanding of UTIs in PLWD.

This study contains a few limitations that would also allow for future research directions. Whilst this work was conducted using readily available and low-cost sensors (with preliminary analysis of sensor importance presented in Supplementary Information Section 15), further directions of work could improve the understanding of the balance between the cost and complexity of deployment and UTI risk prediction performance. The deployed PIR sensors allow us to collect data at low cost, but they do not allow for the distinction between data generated by the person of study and other members of the house. Further work could explore methods of passively collecting personalised data. We found that the sleep mat (which does collect personalised data) significantly improved the analysis performance (Supplementary Information Section 15). In Supplementary Information Section 11, we discuss the model reliability and calibration and find that our model overestimates UTI risk, likely because of the data imbalance in the training set. This motivates the applied risk stratification which allows the monitoring team to balance the sensitivity and specificity with the number of generated alerts; however, when deploying this system, work should be done to understand the trade-off between false positives and false negatives to ensure that the risk groups are well-calibrated. Finally, whilst this work focused on an important section of the population (People Living with Dementia), it would be helpful to apply these techniques in a larger cohort study or one containing older adults in community living environments such as care homes, assisted living, or skilled nursing facilities.

Our feasibility study was conducted within real-world in-home settings on data collected in (near) real-time using off-the-shelf and low-cost sensory technologies and engineered, clinically meaningful, features for predicting UTIs determined by clinicians, urine sample analysis, and a clinical monitoring team. We provide preliminary evidence for the use of such an operation and model which, with further testing, could prove to reduce delays in detecting UTIs in PLWD, and potentially reduce the number of avoidable hospital admissions when used to support clinicians with care. The proposed approach can be scaled rapidly and enable human-in-the-loop decision support by taking advantage of technological advancements, cloud computing, and machine learning. Moreover, risk stratification allows for model calibration to improve patient outcomes and care delivery whilst balancing the cost associated with testing for UTIs. Within an ongoing study or in production, the group thresholds can be modified over time to account for care team resources. SHAP analysis will enable the presentation of explainable results (such as in Supplementary Information Section 13), allowing clinicians to explore why the UTI algorithm has made a given prediction. Additional future work will involve continuing to investigate our in-home monitoring systems’ effects on clinical outcomes, as well as patients’ quality of life.

When deployed, our model will be continually trained on new data as collected. To ensure the performance consistently meets a minimum standard, we will routinely evaluate the model on a test set and track its performance. Feature importance will also be monitored to confirm the algorithm is producing clinically founded results. This will enable rapid debugging of errors and maintain a high level of quality in predictions.

## Methods

### Study design and population

This study was performed in collaboration with Imperial College London and Surrey and Borders Partnership NHS Trust. Participants were recruited from the following: (1) health and social care partners within the primary care network and community NHS trusts, (2) urgent and acute care services within the NHS, (3) social services who oversee sheltered and extra care sheltered housing schemes, (4) NHS Community Mental Health Teams for older adults (CMHT-OP), and (5) specialist memory services at Surrey and Borders Partnership NHS Foundation Trust. All participants provided written informed consent. Capacity to consent was assessed according to Good Clinical Practice, as detailed in the Research Governance Framework for Health and Social Care (Department of Health 2005) and the Mental Capacity Act 2005. Participants were provided with a Participant Information Sheet (PIS) that includes information on how the study used their personal data collected in accordance with the GDPR requirements. If the participant was deemed to lack capacity, a personal or professional consultee was sought to provide written consent to the study. Additionally, capacity of both the participant and study partner is assessed at each research visit. Research staff conducting the assessment have completed the NIHR GCP training and Valid Informed Consent training. If a participant is deemed to lack capacity but is willing to take part in the research, a personal consultee is sought in the first instance to sign a declaration of consent. If no personal consultee can be found, a professional consultee, such as a key worker, is sought. This process is included in the study protocol and ethical panel approval is obtained.

Eligible study participants included adults >50 years with a clinically ascertained diagnosis of dementia or mild cognitive impairment and current or previous treatment at a psychiatric unit. Participants lacking capacity for informed consent were required to have a partner or caregiver who had known them for at least 6 months and was able to attend research assessments with them. Exclusion criteria were as follows: (1) patients receiving treatment for terminal illness (2) presence of severe mental health conditions including depression, anxiety, psychosis, and agitation (3) presence of active suicidal thoughts. In total, 117 participants were selected for participation using the above-mentioned recruitment process.

The cohort characteristics can be seen in Table 2 and a patient disposition is available in Supplementary Information Section 1.

**Table 2:** Characteristics of the study cohort.

Characteristic	Entire cohort		Labelled cohort	
	No.	%	No.	%
**Sex**				
Female	54	46	27	42
Male	63	54	37	58
**Birth year**				
1920-1930	13	11	5	8
1930-1940	47	40	27	42
1940-1950	41	35	26	41
1950-1960	13	11	5	8
1960-1970	2	2	1	2
1970-1980	1	1	0	0
**Ethnicity**				
White	95	81	60	94
Asian	8	7	3	5
Black/African/Caribbean	3	3	0	0
Mixed/Multiple Groups	1	1	0	0
N/A	10	9	1	2
**Household**				
Lives Alone	45	38	16	25
Lives with Partner	60	47		73
N/A	12	10	1	2
**Primary diagnosis**				
Alzheimer’s	61	52	39	61
Vascular Dementia	10	9	5	8
Parkinson’s	5	4	2	3
Other and Mixed	40	34	18	28
N/A	1	1	0	0

Some participants requested not to share their information outside the study and correspond to the Not Available information.

### Data collection and definition of outcome

Demographic data was collected during the baseline assessment, whilst psychometric scales were used to collect various physical and cognitive data during regular visits. In-home observation and measurement data was obtained using low-cost off-the-shelf monitoring technologies, including PIR sensors (for measuring activity) and sleep monitoring devices. Figure 1 presents cohort-wide sleep and activity activations, and differences in sleep and activity for a participant with both UTI positive and negative days. PIR sensors can detect motion within 9 metres and with a maximum angle of 45^∘^ and the sleep mat device can monitor breathing rates, heart rates, and sleep states. For an illustration of the layout of sensors see Supplementary Information Section 2.

Urine samples were collected from several enrolled participants to be labelled by clinicians. Additionally, a baseline algorithm developed in our previous work^40^ suggested patients to the study monitoring team to check for additional symptoms of UTIs and arrange a sample collection and refer to the GP if needed. Once samples were collected, a urine sample analysis was performed and the results sent to clinicians, who with information from the monitoring team, determine a UTI. In total, we have 258 labelled urine samples from 64 participants, of which 81 were confirmed positive UTI cases. If a single day has been labelled, we assume the preceding and proceeding 3 days would also be labelled the same (see Supplementary Information Section 6). This extends the number of labelled days of data to 1752, consisting of 534 positives and 1218 negatives. For our experimentation, we used data collected between 2021/06/28 and 2022/12/01. The models were trained to predict whether a participant had a UTI on a given day (24 h time window). The distribution of labels can be found in Supplementary Information Section 3.

### Data pre-processing and feature selection

In addition to sensor readings, we performed feature engineering inspired by well-known symptoms of UTIs such as incontinence, urgency and increased frequency of urination, and behavioural changes (https://www.nhs.uk/conditions/urinary-tract-infections-utis/) to allow clinical interpretability and improve model performance and generalisability.

Raw features were: (1) frequency of bathroom, bedroom, hallway, kitchen, lounge activations; (2) mean and standard deviation of nocturnal heart rate and respiratory rate; (3) nocturnal awake occurrences. Engineered features were: (4) bathroom day and nocturnal frequencies, moving average, and percentage change; (5) mean and standard deviation of the movement time from any location within the house to bathroom; (6) daily entropy in PIR sensor activation; (7) number of previous UTIs to date. More information on the features selected can be found in Supplementary Information Section 4.

Data collection occurred outside controlled environments using in-home devices so missing measurements inevitably occurred. To limit the effects of incomplete data^41^, we imputed missing values based on strategies depending on the given features (see Supplementary Information Section 5 for more information).

### Analysis platform

All analyses were performed on a secure computing environment at Imperial College London using Python version 3.9. The Pandas^42^, Numpy^43^, Scikit-Learn^44^, and Pytorch^45^ packages formed much of our pipeline.

### Methodology

To ensure generalisability, we evaluated our work in two different ways.

Firstly, the dataset was split temporally into training and testing subsets in an 80:20 ratio. The data collected from 2021/06/28 to 2022/10/05 represented 80% (*n* = 1394 days in total, from *p* = 54 participants) of the dataset, whilst the data between 2022/10/05 and 2022/12/01 represented 20% (*n* = 358, *p* = 39).

This formed the first analysis, evaluating the model at making predictions on future data from the same cohort as it was trained on. We will refer to this experimental setting as “Date Split".

In the second analysis, we used a leave-one-out cross-validation strategy^46^. Here, data was split in the same way as in the first evaluation method. Then, training and testing of our machine learning models was performed using a leave-one-out strategy on data from each of the PLWD. This way, we are able to test the model performance on data from participants outside of the cohort it has been trained on. We will refer to this experimental setting as “Date-ID Split".

During model development and whilst optimising model parameters, validation sets were produced by splitting the training data on the date 2022/09/11. All experiments were performed multiple times, with each run using a bootstrap sample^46^ of the training set to ensure reproducibility. See Supplementary Information Section 8 for a visualisation of this evaluation.

We used sensitivity, specificity, and area under the precision-recall curve to measure model performance (for definitions of metrics, please see Supplementary Information Section 7).

### Model development

We tested Logistic Regression (LR), Extreme Gradient Boosting Decision Tree (XGBoost)^27^, Multilayer Perceptron (MLP), Self-Attention^28^, Random Forest (RF)^29^ and Naive Bayes (NB) models at predicting the risk of UTIs. Hyper-parameters were tuned using Bayesian optimisation on train-validation splits, with the model producing the highest area under the precision-recall curve (on validation data) selected for the final analysis. The number of days of data used as input to the model was jointly tested, ranging from 1 day to 7 days. Supplementary Information Section 9 contains information on the decisions made regarding each step of the UTI model pipeline.

### Stratification of risk scores for clinical reporting

Risk scores from the model are stratified into three groups, used to inform clinical decisions in a concise way and provide precise control over the number of actionable alerts. Outputs are split into the groups Green, Amber, and Red; referring to minimal, medium, and high risk of a UTI respectively. By varying these thresholds, we can balance levels of sensitivity and specificity for the different groups with the number of alerts. This allows our process to be flexible to different clinical scenarios and resources.

Within this work, the optimal thresholds used in our analysis of results were calculated using the algorithm’s predictions on the data collected between 2022/09/11 and 2022/10/05 (validation data), and with feedback from a clinical team. More information on the risk stratification is included in Supplementary Information Section 12.

### Ethics approval

The study received ethical approval from the London-Surrey Borders Research Ethics Committee; TIHM 1.5 REC: 19/LO/0102. The study is registered with National Institute for Health and Care Research (NIHR) in the United Kingdom under Integrated Research Application System (IRAS) registration number 257561.

### Reporting summary

Further information on research design is available in the Nature Research Reporting Summary linked to this article.

### Supplementary information

CR&T Group Members

Supplementary Materials

Reporting Summary

## Data Availability

The data that support the findings of this study are available from the corresponding author upon reasonable request.

## Code availability

The code used in this study will be made available by the corresponding author upon reasonable request.
