# Supplementary material for: Digital remote monitoring for screening and early detection of urinary tract infections
Source: NPJ Digit Med. 2024 Jan 13;7:11. doi: 10.1038/s41746-023-00995-5 (PMC10787784; doi:10.1038/s41746-023-00995-5)
Supplement: Supplementary file 1 — CR&T Group Members [file 41746_2023_995_MOESM1_ESM.pdf]

CR&T Group

Acknowledgement list for UK Dementia Research Institute (UK DRI) Care Research & Technology (CR&T) Centre publications using the Minder core data set. The primary contact for this group is: `ukdri.crt@imperial.ac.uk`

Leadership and Management: David Sharp (Director), Danielle Wilson (Centre Manager), Sarah Daniels (Health and Social Care Lead), David Wingfield (General Practice Lead), Matthew Harrison (Human-Centred Design Lead), Shlomi Haar (Movement and Living Lab Lead), Mara Golemme (Project Manager).

Behaviour and Cognition Group: David Sharp (Group Lead), Martina Del Giovanne, Paresh Malhotra, Neil Graham, Emma Jane Mallas, Naomi Hassim, Greg Scott, Magdalena Kolanko, Alina-Irina Serban, Helen Lai, Eyal Soreq, Lucia M Li, Tong Wu, Thomas Parker.

Bioelectronic Systems Group: Timothy Constandinou (Group Lead), Alan Bannon, Danilo Mandic, Charalambos, Adrien Rapeaux, Ghena Hammour, Ian Williams, Byran Hsieh, Maowen Yin, Niro Yogendran.

Robotics and AI Interfaces Group: Ravi Vaidyanathan (Group Lead), Ting Su, Maria Lima, Thomas Martineau, Mayue Shi, Melane Jouaiti, Tianbo Xu, Maitreyee Wairagkar, Bo Xiao, Carlot Sebastian Castillo, Alehandro Valdunciel, Panipat Wattansiri, Reineira Seeamber, Annika Guez, Zehao Liu, Saksham Dhawan.

Translational Machine Intelligence Group: Payam Barnaghi (Group Lead), Nan Fletcher-Lloyd, Amer Marzuki, Hamed Haddadi, Francesca Palermo, Mark Woodbridge, Anna Joffe, Yuchen Zhao, Samaneh Kouchaki, Alexander Capstick, Yu Chen, Tianyu Cui.

Point of Care Diagnostics Group: Paul Freemont (Group Lead), Loren Cameron, Thomas Adam, Michael Crone, Raphaella Jackson, Kristen Jensen, Martin Tran.

Sleep and Circadian Group: Derk Jan Dijk (Group Lead), Anne Skeldon, Vikki Revell, Kevin Wells, Giuseppe Atzori, Ullrich Bartsch, Lucina Grainge, Ciro Della Monica, Hana Hassanin, Kiran GR Kumar, James Woolley, Damion Lambert, Iris Wood-Campar, Sara Mohammadi Mahvash, Janetta Rexha, Thalia Rodrigues Garcia.

Movement and Movement Disorders Group: Shlomi Haar (Group Lead), Subai Abulikemu, Niro Yogendran, Julian Jeyasingh Jacob, Cosima Graef, Nathan Steadman, Akena Kutuzova, Federico Nardi, Assaf Touboul.

Human Centred Design Group: Matthew Harrison (Group Lead), Lenny Naar, Sophie Horrocks, Brian Quan.

Site Investigators and Key Personnel: Ramin Nilforooshan (Chief Investigator), Jessia True (Research), Olga Balazikova (Research), Emily Beal (Research Co-ordinator), Chloe Walsh, Nicole Whitethread, Matthew Purnell, Vaiva Zarombaite, Lucy Copps, Olivia Knight, Gaganpreet Bangar, Sumit Dey, Chelsea Mukonda, Jessia Hine, Luke Mallon (Clinical Monitoring Team), David Winfield (Principal Investigator), Claire Norman (Research Nurse), Aanesha Patel, Ruby Lyall, Sanara Razall (Clinical Studies), Naomi Hassim, Pippa Kirby, John Patterson (Research Therapists), Mike Law (Business Development), Andy Kenny (Social Services).
