# Supplementary material for: Digital remote monitoring for screening and early detection of urinary tract infections
Source: NPJ Digit Med. 2024 Jan 13;7:11. doi: 10.1038/s41746-023-00995-5 (PMC10787784; doi:10.1038/s41746-023-00995-5)
Supplement: Supplementary file 2 — Supplementary Materials [file 41746_2023_995_MOESM2_ESM.pdf]

Supplementary Materials

1 Participant Disposition

Supplementary Figure 1 shows the number of participants and days of data that were used within each stage of the analysis.

2 Sensor Layout

Supplementary Figure 2 illustrates an example of the layout of sensors within a home.

3 Distribution of Labels

The composition of the dataset can be seen in Supplementary Figure 3, which demonstrates the wealth of data available within the study. We also see that the vast majority of participants have few UTI labels, reducing the bias from overfitting during model training.

4 Feature Information

The raw features used in our predictive algorithm were:

- Frequency of Bathroom, Bedroom, Hallway, Kitchen, Lounge rooms: the number of times that a person enters in a specific room over a day.
- Mean and Standard Deviation of Night-time Heart Rate and Respiratory Rate: mean and standard deviation extracted from the heart rate and respiratory rate sensors within the bed mattress.
- Night-time Awake Occurrences: the number of times a person is awake on the bed mattress during the night.

The engineered features calculated for input into the model are:

- Bathroom Day and Night-time Frequencies, Moving Average, and Percentage Change: the number of times a person accesses the bathroom during the morning/afternoon and the night, as well as the moving average and percentage change.
- Daily Entropy: The entropy rate, calculated on a transition matrix of all consecutive PIR sensor activation in a single day. This is a measure of the randomness of a person's activity patterns.
- Mean and Standard Deviation of the movement time from locations in the house to Bathroom: mean and standard deviation of the time required to move to the bathroom from the previous location. We use this as a measure of the person's urgency to urinate.
- Number of Previous UTIs to Date: the number of times a person has suffered from UTI before the investigated date.

5 Data Imputation

Each raw PIR sensor data point contains a household identifier, date and time, and the sensor that was triggered. To pre-process this data, we first calculate the frequency of each sensor's activations during a day. However, since not all sensor data is available due to device or network issues we impute sensor activation for the missing frequencies. To do this, we assume that each of the PLWD do not have a given sensor installed until it has been active at least once before, at which point those future values are imputed with 0 (instead of N/A). From these values, all of the activity based features are calculated.

Each night time physiology sensor data point is streamed with a PLWD identifier, date and time, the sleep state (deep, REM, light, awake), and the heart rate and respiratory rate measurement. To pre-process this data, we calculate the frequency of each sleep state during a day, and the mean and standard deviations of the heart and respiratory rates.

After these values are calculated, if missing values still persist, we impute them using the mean values for that feature in the cohort. We also experimented with imputing missing values based on age-matched data and data from each individual's history. However, these methods did not perform as well.

6 Labelling Days Either Side of Sample Collection

Within our analysis, when training and testing our machine learning pipeline, we have extended the number of labelled days of data by considering that the 3 days preceding and proceeding a sample collection date (where the sample was later labelled as positive or negative UTI) have the same label. This increases the number of labelled days of data, but also allows us to balance the distribution of days in the week that are labelled. The sample collection and testing in our study operates on a weekly basis, creating a distribution as shown in Supplementary Figure 4a. If these labels were used for the single day of obtaining the clinical UTI test results, the trained machine learning model would likely be biased based on the recording date. Extending the data by 3 days on either side of the recorded test result has the added benefit of balancing the labels equally over all days of a week, allowing for more variation in the training data. The new distribution can be seen in Supplementary Figure 4b.

7 Evaluation Metrics

UTI is a common infection in older adults and in particular PLWD. UTIs can result in serious complications and hospitalisation. In this study, we have proposed machine learning models to predict the risk of a UTIs. However, evaluating their performance is essential to ensure their reliability and usefulness in clinical practice. In this section, we discuss the evaluation metrics used to assess the performance of the proposed machine learning models.

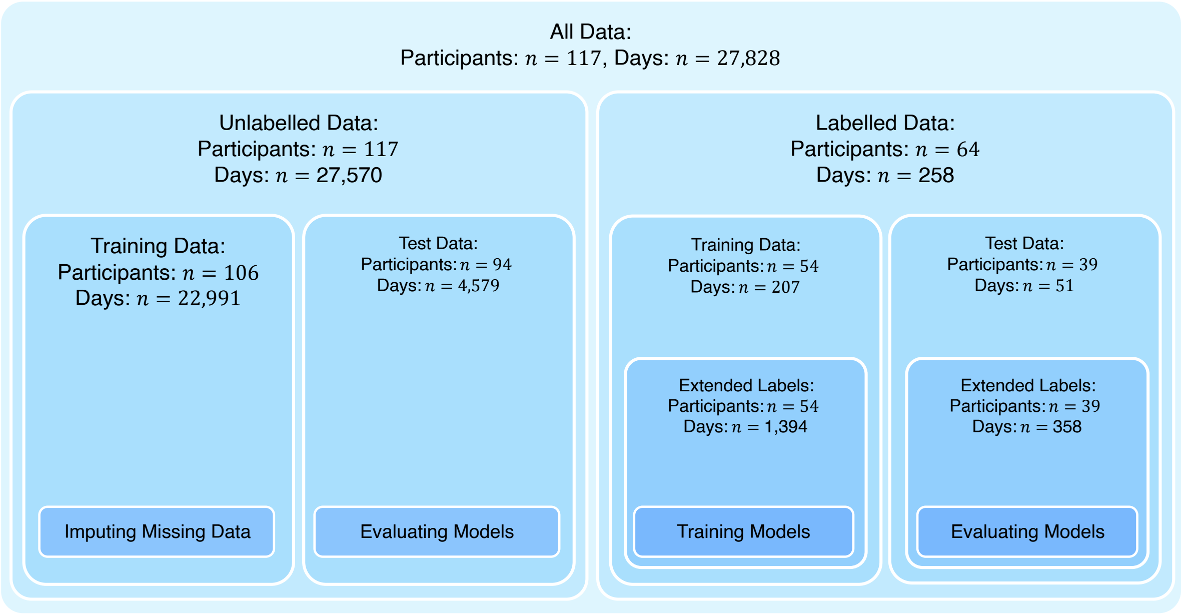

Supplementary Figure 1: Patient disposition for the imputing, training, and evaluating stages of our analysis.

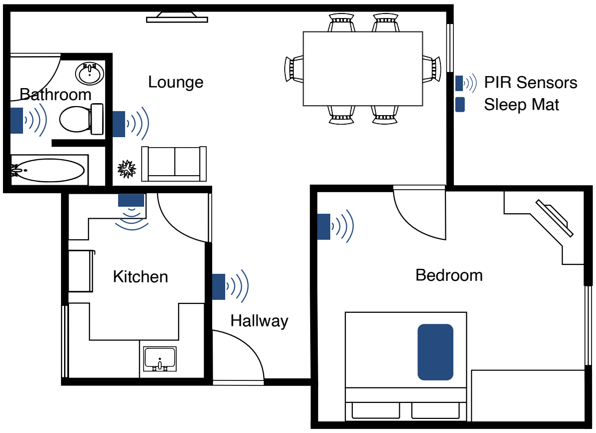

Supplementary Figure 2: Illustration of sensor deployment within the home of a PLWD. Here, PIR sensors are located in each room for in-home activity monitoring. A sensor is located under the mattress for analysing physiology and movements in and out of the bed. All sensors collect data passively.

Three evaluation metrics were used in our study, including specificity, sensitivity, and area under the precision-recall curve (AUC Precision-Recall). Each metric provides important information about the performance of the model, and their combined use helps provide a comprehensive picture of the model's predictive ability.

Sensitivity (and equivalently, recall) is the proportion of true positive predictions among all actual positive cases. It measures the model's ability to correctly identify individuals who are at risk of developing a UTI. Specificity, on the other hand, measures the model's ability to correctly identify individuals who are not at risk of a UTI. A high sensitivity indicates that the model is effective at identifying those who are at risk of a UTI, while a high specificity indicates that the model is effective at identifying those who are not at risk.

$$\text{Sensitivity} = \frac{TP}{TP + FN}$$

$$\text{Specificity} = \frac{TN}{FP + TN}$$

Where TP, TN, FP, and FN refer to True Positives, True Negatives, False Positives, and False Negatives, respectively.

The AUC precision-recall is another commonly used metric that provides an overall measure of the performance of a model. It is defined as the area under the curve created by plotting the precision of a model against its recall, as the thresholds on the positive prediction are varied. It is preferred over metrics such as area under the receiver operating characteristic curve (which plots the true positive rate against the false positive rate at various thresholds) when the labels in the dataset are imbalanced.

In summary, evaluating the performance of machine learning models for detecting the risk of UTI events using in-home monitoring data requires the use of multiple evaluation metrics. The sensitivity, specificity, and area under the precision-recall curve provide important information about the model's ability to identify those at risk of a UTI and the overall performance. Understanding these metrics can help healthcare providers to assess the reliability and usefulness of these models in clinical practice.

8 Evaluation Procedures

When evaluating our models, we chose to use two different methods as described in the Methodology Section. Supplementary Figure 5 illustrates these procedures. The difference between these methods is clear when performing the final testing, in which "Date-ID Split" uses a Leave-One-Out strategy on the IDs to measure performance. In "Date-ID Split", metrics are calculated over the combined predictions over all of the PLWD.

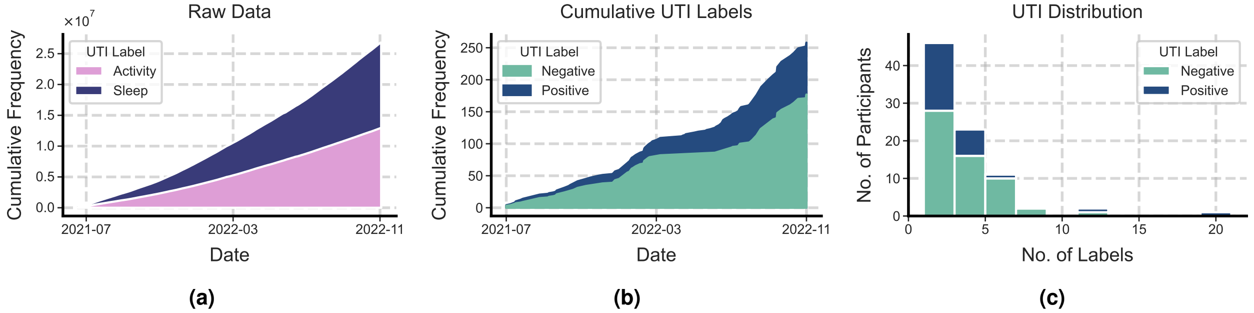

Supplementary Figure 3: Dataset characteristics. **a** The cumulative number of sleep and activity data points recorded in total. **b** The cumulative number of clinically verified UTI labels over time. **c** The distribution of UTI labels by participant. We see that the vast majority of participants have few verified labels, which will reduce bias from overfitting in the model.

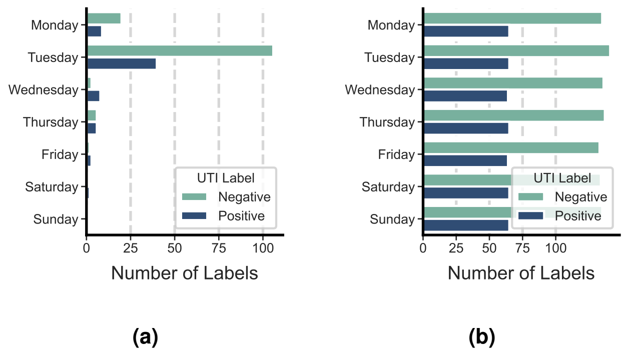

Supplementary Figure 4: Label distribution by day. **a** The days for which we have verified UTI labels. **b** The distribution of these labels after they are extended by 3 days either side.

9 Machine Learning Pipelines Evaluated

Before arriving at our final machine learning pipeline, we evaluated several combinations of imputing strategies, scaling techniques, and models, as well as the number of days used as inputs to the models. In this section, we describe the different techniques that we experimented with.

Imputation Strategies

After imputing values based on intuition (for example, missing activity implies zero activity: see Appendix 5), the dataset still contains some missing values that need to be imputed before applying machine learning models. The following are the techniques that we tested:

- **Global Imputation** required using all of the data from the training sets (unlabelled and labelled data) to impute the missing values in the training, validation, and test data sets. When doing this, we imputed values using the mean of a given feature's values.
- **Age Matched Imputation** was based around ensuring that data was imputed with age-matched background data. When imputing missing values from the training, validation, or testing data sets, unlabelled and labelled training data were filtered to ensure that a given point was being imputed based on the data from the most n similar ages present in the data. We set $n = 15$ in our experiments. This was decided based on the size and the age distribution in the cohort to provide sufficient and balanced number of similar matches. After the background data was chosen, we used three different approaches to perform the imputation:
  - Using the feature means.
  - Based on a K-Neareset Neighbour strategy.[1]
  - Based on an iterative approach using a Random Forest Regression model.[2]

  Ultimately, we found that applying age-matched imputation performed worse than global imputation, and required higher compute costs, limiting its use in production.

Input Scaling

After the data is imputed, but before it is passed to a machine learning model, the data must be scaled to ensure that features are weighted equally when training. To do this, we tested two methods of data scaling:

- **Global Scaling** involved using the mean and standard deviation of each feature from training data across the cohort to normalise the training, validation, and testing data sets so that the training data had a mean of zero and standard deviation of one in each feature. The same scaling parameters were then used to transform the validation and testing sets to ensure no data leakage.
- **PLWD Scaling** involved calculating the mean and standard deviation on the training data for each feature and each of the PLWD separately and using these to scale the training, validation, and testing data for the same PLWD. This was done in an effort to reduce the model's ability to learn to predict PLWD who often had UTIs rather than learning representations of days of UTI positive and negative. When PLWD were present in the validation and testing sets but not in the training data, global statistics were used to scale their data. However, this proved to be unfeasible in practice since it meant that participants were not being consistently scaled in the same way, and when applying different data splits, the same participants were being scaled differently.

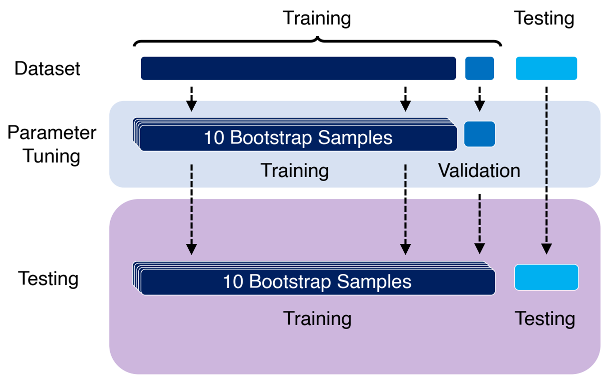

(a) Date Split

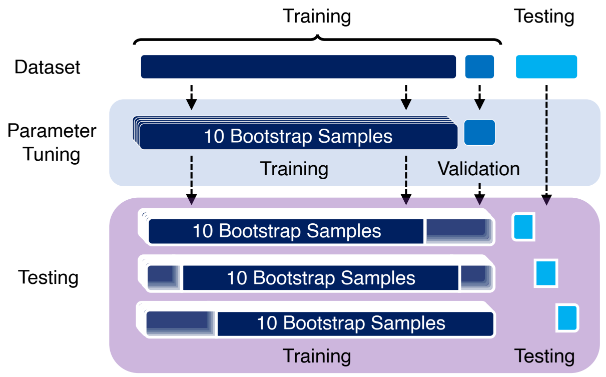

(b) Date-ID Split

Supplementary Figure 5: The evaluation procedures visualised. a “Date Split”. **b** “Date ID Split” with the Leave-One-Out evaluation acting over the PLWD.

Machine Learning Models

After data is pre-processed, various machine learning models from deep learning and conventional machine learning methods were evaluated on their performance at predicting days labelled as a positive or negative UTI. We examined the following:

- Logistic Regression (LR): With L2 regularisation, with a value in $[0.01, 1]$ determined by hyper-parameter optimisation.
- Extreme Gradient Boosting (XGBoost):[3] With a learning rate between $[0.01, 0.5]$, number of estimators between $[50, 500]$, max tree depth of $[1, 10]$, sub-sample ratio between $[0.01, 1]$, and L1 regularisation between $[0, 0.001]$ determined by hyper-parameter optimisation.
- Multilayer Perceptron (MLP): With layer sizes of $(M, 50, 50, 2)$ (with M the number of features), ReLU activation function, batch size of 200, max number of epochs of 500, Adam optimiser[4] with $\beta_1 = 0.9$ and $\beta_2 = 0.999$, an L2 regularisation between $[0, 0.001]$, and a learning rate between $[0.001, 0.01]$ determined by hyper-parameter optimisation.
- Self-Attention (S-Attn) model:[5] A transformer-encoder style model with 5 self-attention heads, fully connected layer sizes of 100, max number of epochs of 100, Adam optimiser[4] with $\beta_1 = 0.9$ and $\beta_2 = 0.999$, an L2 regularisation between $[0, 0.001]$, and a learning rate between $[0.001, 0.01]$ determined by hyper-parameter optimisation.
- Random Forest (RF):[6] With Gini entropy, number of estimators between $[50, 500]$ and a max depth between $[1, 100]$ determined by hyper-parameter optimisation.
- Gaussian Naive Bayes (NB): With variance smoothing of 1×10^{-9}.

These models were run using Python, and were implemented using Scikit-Learn[7] and Pytorch.[8]

10 Results of All Models Tested

Table 1 presents the results from all of the experiments using the imputation based on the mean of the features, and the global scaling.

11 Reliability and Calibration

This section presents the results of the model’s reliability and calibration analysis.

Supplementary Figure 6 shows the reliability plot for the model, which presents the agreement between the model risk scores and the observed accuracy[9–11] on the test set (calculated over both the positive and negative UTI predictions). This shows that the risk scores can be considered reliable when considering both positive UTI and negative UTI predictions together. This is seen by observing the small gaps in the top plot and the distance between the average accuracy and average confidence in the lower plot.

However, when considering only the positive UTI predictions, Supplementary Figure 7 shows that the best-performing model was over-confident at times, a common characteristic of models trained within environments with significant weight imbalance. This has been a key motivation of designing and applying the risk stratification technique, to ensure that the number of false positives can be controlled in line with the resources and requirements of the care team.

12 Risk Stratification Analysis

Previous work on predicting the occurrence of UTIs in populations have presented results as risk scores or definitive predictions of positive or negative UTI. However, since we consider our model to be used as a tool for clinicians to better understand patients within their care and as an alerting system, we believe a more advisory approach is required. To this end, when reporting our predictions to clinicians, we stratify results into three levels of alert; Green, Amber, and Red. Green, Amber, and Red aim to represent minimal, medium, and high risk of UTI respectively. We expect a Green alert to require no action, an Amber alert to possibly require further consideration from the clinician and analysis of in-home behaviour and medical history, and a Red alert to suggest that further analysis of symptoms or testing is performed.

Once a machine learning algorithm has been trained on the training data and the best performing model is chosen, risk scores are calculated on the validation data so that they can be used to determine the thresholds for the final model. We

Table 1: Mean (95% CI) % of the AUC Precision-Recall of the different models tested with the chosen imputation and scaling methods with 10 bootstrap repeats. The bold row shows the method achieving the best result on the "Date-ID Split" and the chosen model for our final pipeline.

		Date-ID Split		Date Split	
Model	No. of Days	Validation	Test	Validation	Test
LR	1	75.6 (73.6 - 77.5)	65.3 (63.6 - 67.0)	65.8 (64.0 - 67.7)	56.8 (55.7 - 57.9)
LR	2	76.0 (72.4 - 79.7)	61.9 (60.8 - 63.0)	67.6 (66.4 - 68.7)	55.2 (54.4 - 56.0)
**LR**	**3**	**78.3 (76.8 - 79.7)**	**63.5 (61.8 - 65.2)**	**67.7 (66.4 - 69.0)**	**54.4 (53.4 - 55.4)**
LR	4	76.0 (72.8 - 79.1)	63.5 (62.5 - 64.6)	64.2 (61.7 - 66.8)	55.9 (55.0 - 56.8)
LR	5	73.9 (67.2 - 80.6)	61.9 (61.1 - 62.6)	66.0 (63.8 - 68.3)	56.8 (55.8 - 57.9)
LR	6	72.6 (64.0 - 81.2)	63.5 (61.9 - 65.1)	73.1 (70.6 - 75.7)	57.0 (55.8 - 58.1)
LR	7	69.8 (62.6 - 77.0)	65.4 (64.5 - 66.3)	70.3 (67.8 - 72.7)	58.4 (57.4 - 59.5)
MLP	1	40.6 (30.1 - 51.0)	37.2 (28.2 - 46.2)	65.9 (63.8 - 68.1)	48.5 (45.5 - 51.5)
MLP	2	42.9 (29.6 - 56.2)	35.5 (28.0 - 43.0)	64.2 (62.7 - 65.7)	53.1 (49.8 - 56.3)
MLP	3	48.2 (33.3 - 63.1)	35.9 (26.0 - 45.7)	61.8 (58.4 - 65.2)	54.6 (52.2 - 56.9)
MLP	4	48.0 (36.3 - 59.7)	32.8 (24.3 - 41.3)	62.7 (60.5 - 64.8)	53.3 (50.2 - 56.3)
MLP	5	49.0 (40.3 - 57.6)	30.8 (23.7 - 37.9)	62.7 (60.5 - 65.0)	54.0 (50.3 - 57.7)
MLP	6	46.2 (34.2 - 58.2)	29.8 (20.0 - 39.5)	63.9 (59.5 - 68.4)	59.2 (54.9 - 63.5)
MLP	7	48.5 (37.0 - 60.1)	31.3 (18.7 - 43.9)	63.1 (57.7 - 68.5)	53.5 (49.6 - 57.5)
NB	1	69.7 (69.0 - 70.5)	68.1 (66.4 - 69.7)	63.9 (63.2 - 64.6)	59.0 (57.3 - 60.6)
NB	2	63.6 (60.4 - 66.8)	66.2 (65.0 - 67.5)	61.6 (59.5 - 63.8)	58.5 (57.1 - 59.9)
NB	3	58.4 (56.1 - 60.8)	60.7 (57.3 - 64.0)	54.5 (53.9 - 55.0)	58.6 (57.0 - 60.2)
NB	4	60.5 (58.4 - 62.6)	59.8 (58.3 - 61.4)	54.4 (53.0 - 55.8)	57.4 (55.1 - 59.7)
NB	5	64.6 (61.8 - 67.3)	58.9 (58.2 - 59.7)	58.1 (56.5 - 59.8)	56.8 (56.1 - 57.5)
NB	6	68.8 (65.2 - 72.4)	59.8 (58.6 - 61.0)	63.1 (61.1 - 65.1)	56.4 (55.5 - 57.3)
NB	7	72.3 (67.5 - 77.1)	59.4 (58.4 - 60.5)	68.3 (66.4 - 70.1)	55.0 (54.0 - 56.1)
RF	1	25.7 (21.4 - 30.0)	27.6 (26.0 - 29.1)	61.4 (60.3 - 62.5)	68.3 (66.7 - 69.9)
RF	2	26.3 (19.0 - 33.5)	30.6 (28.2 - 33.0)	61.6 (60.2 - 63.0)	70.2 (68.4 - 72.1)
RF	3	30.3 (27.2 - 33.4)	32.2 (28.3 - 36.0)	60.2 (58.4 - 61.9)	70.2 (68.5 - 72.0)
RF	4	25.1 (22.2 - 28.1)	32.2 (28.2 - 36.1)	57.7 (56.4 - 59.0)	72.5 (70.7 - 74.2)
RF	5	23.5 (21.2 - 25.8)	27.1 (25.3 - 28.9)	55.4 (53.5 - 57.2)	70.9 (68.7 - 73.1)
RF	6	23.3 (20.3 - 26.2)	25.6 (22.8 - 28.3)	55.8 (53.0 - 58.6)	73.0 (70.9 - 75.0)
RF	7	26.3 (22.5 - 30.1)	23.6 (21.9 - 25.3)	55.0 (53.1 - 57.0)	74.4 (72.0 - 76.8)
S-Attn	1	52.2 (42.9 - 61.4)	45.1 (38.4 - 51.8)	59.0 (54.7 - 63.3)	53.6 (50.7 - 56.4)
S-Attn	2	47.4 (34.0 - 60.7)	37.9 (29.8 - 46.0)	51.6 (45.0 - 58.2)	56.0 (52.2 - 59.7)
S-Attn	3	48.5 (37.7 - 59.4)	34.8 (25.8 - 43.8)	56.6 (53.8 - 59.4)	53.8 (50.9 - 56.6)
S-Attn	4	49.3 (36.6 - 62.0)	39.2 (31.4 - 46.9)	55.5 (51.6 - 59.4)	57.2 (53.4 - 61.1)
S-Attn	5	37.4 (28.2 - 46.6)	37.3 (33.0 - 41.6)	50.7 (44.4 - 57.0)	54.8 (49.3 - 60.2)
S-Attn	6	35.9 (25.7 - 46.0)	35.4 (27.9 - 42.8)	48.9 (42.1 - 55.7)	51.9 (47.0 - 56.8)
S-Attn	7	53.6 (35.0 - 72.2)	27.7 (22.6 - 32.9)	48.0 (38.7 - 57.3)	48.8 (42.8 - 54.7)
XGBoost	1	23.8 (21.5 - 26.1)	28.7 (26.0 - 31.5)	61.2 (59.5 - 62.8)	63.7 (62.4 - 65.1)
XGBoost	2	23.5 (20.6 - 26.4)	31.5 (27.3 - 35.8)	57.7 (53.8 - 61.6)	65.4 (63.4 - 67.5)
XGBoost	3	24.3 (18.5 - 30.1)	36.5 (33.2 - 39.8)	57.2 (53.8 - 60.6)	58.5 (55.7 - 61.3)
XGBoost	4	21.1 (19.0 - 23.1)	29.3 (27.8 - 30.7)	57.5 (56.7 - 58.3)	70.6 (68.3 - 72.8)
XGBoost	5	21.0 (19.1 - 22.8)	32.8 (29.2 - 36.4)	52.7 (49.8 - 55.7)	52.8 (47.8 - 57.9)
XGBoost	6	22.0 (20.2 - 23.7)	26.1 (23.5 - 28.8)	52.7 (49.8 - 55.6)	71.4 (68.7 - 74.1)
XGBoost	7	19.7 (17.6 - 21.7)	26.5 (24.8 - 28.2)	53.3 (51.3 - 55.4)	68.4 (66.2 - 70.7)

use the verified UTI labels on the validation set, alongside the risk scores to calculate the sensitivity and specificity of stratified groups under different threshold values. These metrics need to be considered in addition to the number of Red alerts raised to ensure that the algorithm is tuned to resources of the deployed environment. Thresholds that produce high sensitivity might not be viable within a given environment due to a high number of alerts (and maybe high false positive rates).

When investigating the best thresholds, our goal was to maximise the sensitivity and specificity, whilst ensuring that the number of generated alerts was realistic considering the prevalence of UTIs, risk, and the available resources. To achieve this in our example of thresholds, we varied the thresholds of the stratification with a resolution of 10% and computed the sensitivity and specificity of the predictions on the Green and Red groups (assuming Green and Red refer to negative and positive UTI prediction respectively). We optimised these thresholds with the following criteria:

- Jointly maximise sensitivity and specificity, by optimising for their sum:

$$\text{Sensitivity} + \text{Specificity}$$

- Ensure that the rate of Red, R_r alerts on the validation dataset is restricted to the following (corresponding to the overall rate of UTIs in older adults[12]):

$$10\% \leq R_r \leq 15\%$$

After employing these restrictions, we calculated that the most optimal thresholds to apply to the risk score (with range $[0, 100]$) were: Green $\in [0, 30)$, Orange $\in [30, 80)$, and Red $\in [80, 100)$.

The next section shows how the sensitivity and specificity can be varied by changing these thresholds.

12.1 Performance of Varying Thresholds

Supplementary Figure 8 shows how the sensitivity and specificity on the validation set varies as the thresholds on the red and the groups are changed. By studying this figure, thresholds can be chosen to balance the metrics against the number of alerts raised in a cohort. We can see that the specificity is more sensitive to the thresholds than the sensitivity, which is due to the model predicting proportionally more false positives than false negatives.

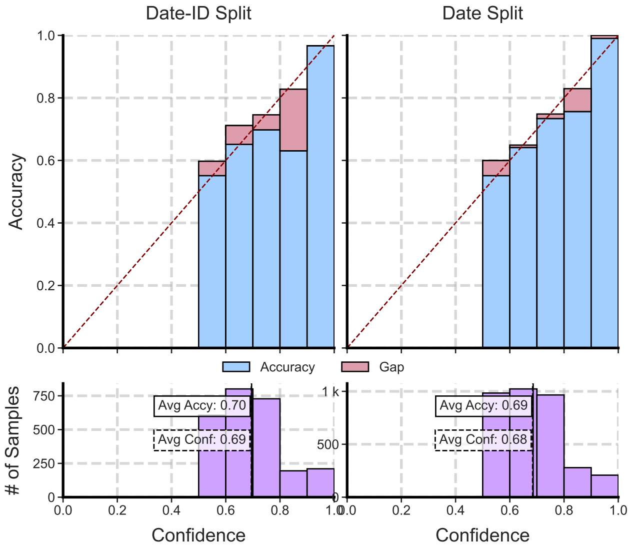

Supplementary Figure 6: Reliability plot. Top shows the model confidence (on positive and negative UTI cases) against accuracy on the test set of “Date Split” and “Date-ID Split”. The gap shows the difference between the average accuracy and confidence of a bin, which would ideally be 0. Bottom shows the histogram of confidences reported by the model on the test set of “Date Split” and “Date-ID Split”.

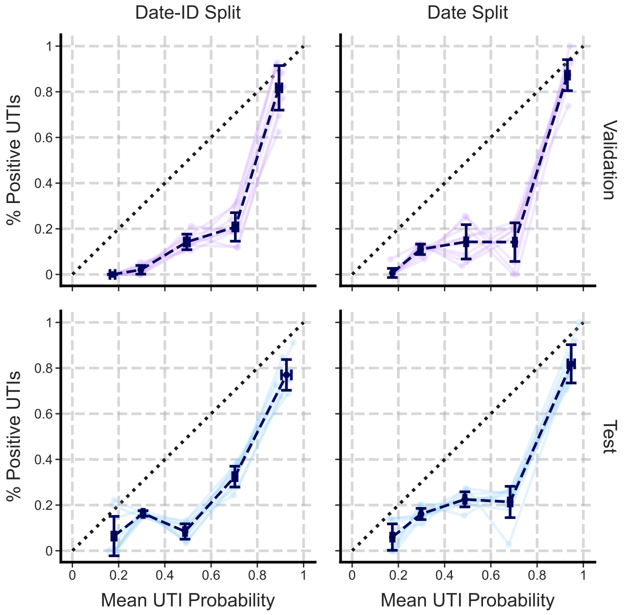

Supplementary Figure 7: Calibration plot. The mean UTI predicted risk against the proportion of positive UTI cases for the best model. This is plotted for the validation and test set from the “Date Split” and “Date-ID Split” data. The error bars represent the standard deviation of the values from the 10 bootstrap repeats.

13 Risk Breakdown by Feature

Supplementary Figure 9 shows four different UTI predictions broken down by SHAP score contributions from each feature. Visualising the predictions like this allows clinicians to explore based on what observations predictions were made.

For example, Supplementary Figure 9a shows that for this single prediction, a large standard deviation of the night time respiratory rate was the largest contributor to the positive prediction, whilst a negative relative change in the moving average of night time bathroom use was indicative of a negative prediction.

Supplementary Figure 10 also shows the feature contribution to a correct positive UTI prediction, layered over a floor plan of a house for improved visualisation and clinical understanding.

14 Examples of Risk Score Over Time

Supplementary Figure 11 shows how the risk score calculated by the best model varies over time on the testing data set of the “Date-ID Split”. We have overlayed the stratification groups and UTI diagnoses for further context.

15 Performance with Fewer Sensors

In this section, we present the performance of the proposed algorithm on data collected using only a subset of the sensors. Here, we have used the same methodology but trained and tested the model on data relating to the PIR sensors and the Sleep sensors only. Table 2 shows the results of this analysis, in which we observe that using the PIR sensors in addition to the sleep sensors significantly improves the overall performance of the predictive model.

These results allow us to reference how the model could perform if sensors are omitted for cost-saving reasons or because of specific deployment requirements.

16 Recurrent UTIs

Within this section we will look at the performance of the model when making predictions on participants who had a UTI at varying numbers of days before the test set.

Supplementary Figure 12 shows the distribution of positive UTI labels in days up to the test set. We can see that the majority of UTI labels in our dataset were measured within 6 months of the test set. Additionally, if we view the number of days between the start of the test set and the most recent positive UTI label per participant, we see a greater skew towards recent positive UTI diagnosis.

To produce the results shown in Supplementary Figure 13a, we matched each prediction in the test set of the “Date Split” with the number of days since their most recent UTI diagnosis. We then calculated the model’s accuracy when making predictions on participants in which the model has not seen a positive UTI example for the given length of time. To understand the performance of the model when predicting UTIs from participants that it has never seen an example for, we need to study the “Date-ID Split” results presented in the Results Section.

Although the model has never seen examples of UTI for these participants (since this is on the “Date-ID Split” test set) Supplementary Figure 13b shows the results of the model split by par-

Table 2: Mean (95% CI) % of sensitivity, specificity, and area under the precision-recall curve of the UTI prediction model on the different feature subsets with 10 bootstrap repeats.

	Feature Subset	Sensitivity	Specificity	AUC Precision-Recall
Date	Activity	**62.6 (60.8 - 64.4)**	64.8 (63.3 - 66.2)	41.4 (40.1 - 42.6)
	Sleep	12.4 (10.7 - 14.2)	66.4 (65.3 - 67.5)	21.8 (21.2 - 22.3)
	All	54.5 (52.7 - 56.4)	**73.0 (71.2 - 74.8)**	**54.4 (53.4 - 55.4)**
Date-ID	Activity	32.0 (29.0 - 34.9)	62.4 (60.8 - 64.0)	36.4 (35.0 - 37.8)
	Sleep	12.5 (11.6 - 13.3)	**72.9 (71.7 - 74.1)**	26.3 (25.8 - 26.9)
	All	**65.3 (64.3 - 66.2)**	70.9 (68.6 - 73.1)	**63.5 (61.8 - 65.2)**

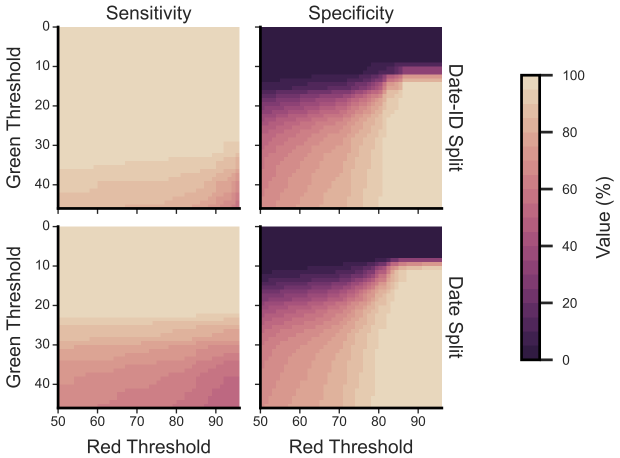

Supplementary Figure 8: Metrics for different thresholds. The sensitivity and specificity of the model on the “Date Split” and “Date-ID Split” validation sets as the green and red thresholds are varied. Here, the results are coloured by the result rounded to the nearest 5% for improved reading.

ticipants with different numbers of previous positive UTIs. Within our dataset the number of positive and negative labels in the test set is imbalanced for these groups, making it hard to interpret the performance of the model on each. Further work, with a larger cohort study, will be done to explore this in more detail.

17 Performance on Female/Male Split

Here we present the performance of the model on the Male and Female participants within our dataset. To do this, we split the predictions made by the proposed model (with thresholds of $> 50\% =$ Positive and $< 50\% =$ Negative) on the test set into those made on PLWD from the Male and Female demographics and then calculate the mean and standard deviation of the accuracy on each of the participants. This can be seen in Table 3 alongside the number of participants in each group and the UTI label proportions.

This experiment highlighted a discrepancy in the accuracy according to the sex of the participants. This is due to the difference in the base rates of labelled positive and negative examples for each sex. We see that Female participants are more likely to contract a UTI than Male participants in our data, agreeing with clinical observations.[13]

A disparity in metrics on different demographics is inevitable when their proportion of labels differs [1]. This should be considered when deploying this screening tool, and if there is sufficient data, a separate model could be trained to make predictions on each sex. However, for this proof-of-concept study, training a separate model for Male and Female participants would lead to overfitting in the model.

If a single model is desired, risk score thresholds could also be tuned on individual sexes with a given fairness metric in mind.[14] For example, Table 3 shows the likelihood of a positive UTI prediction ($\hat{Y} = 1$) by sex using our proposed model (with thresholds of $> 50\% =$ Positive and $< 50\% =$ Negative): $\Pr(\hat{Y} = 1|\text{Sex})$. This is referred to as demographic (or statistical) parity.

Here we see that the model has a high demographic parity as the likelihood of a positive prediction across demographics is close to equal. Varying this threshold would cause either increased false negatives for the Female participants, or increased false positives for the Male participants.

Other fairness metrics can be optimised by changing these thresholds to suit the setting.

[1] https://pair.withgoogle.com/explorables/measuring-fairness/

References

[1] Beretta L, Santaniello A. Nearest neighbor imputation algorithms: a critical evaluation. BMC Medical Informatics and Decision Making. 2016 Jul;16(S3). Available from: https://doi.org/10.1186/s12911-016-0318-z.

[2] Hong S, Lynn HS. Accuracy of random-forest-based imputation of missing data in the presence of non-normality, non-linearity, and interaction. BMC Medical Research Methodology. 2020 Jul;20(1). Available from: https://doi.org/10.1186/s12874-020-01080-1.

[3] Chen T, Guestrin C. XGBoost: A Scalable Tree Boosting System. In: Proceedings of the 22nd ACM SIGKDD International Conference on Knowledge Discovery and Data Mining. KDD ’16. New York, NY, USA: ACM; 2016. p. 785-94. Available from: http://doi.acm.org/10.1145/2939672.2939785.

[4] Kingma DP, Ba J. Adam: A Method for Stochastic Optimization. In: Bengio Y, LeCun Y, editors. 3rd International Conference on Learning Representations, ICLR 2015, San Diego, CA, USA, May 7-9, 2015, Conference Track Proceedings; 2015. Available from: http://arxiv.org/abs/1412.6980.

[5] Vaswani A, Shazeer N, Parmar N, Uszkoreit J, Jones L, Gomez AN, et al. Attention is All You Need. In: Proceedings of the 31st International Conference on Neural Information Processing Systems. NIPS’17. Red Hook, NY, USA: Curran Associates Inc.; 2017. p. 6000–6010.

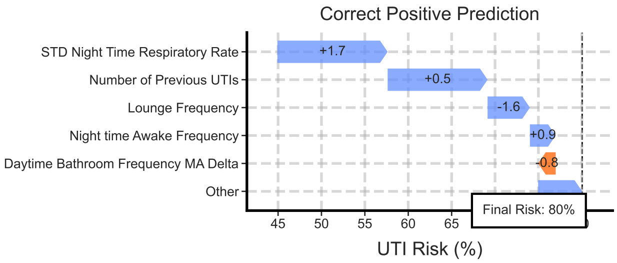

(a) Correct positive prediction

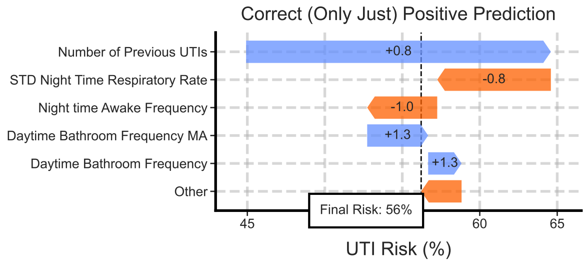

(b) Only just correct positive prediction

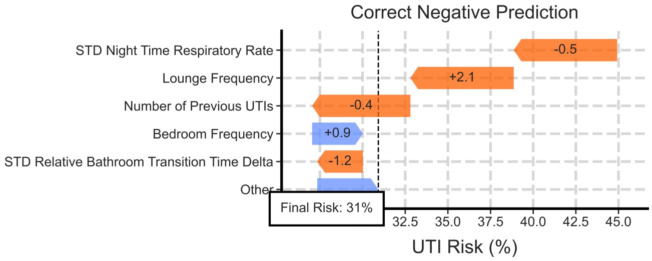

(c) Correct negative prediction

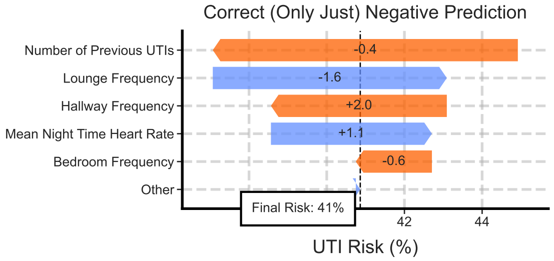

(d) Only just correct negative prediction

Supplementary Figure 9: SHAP values for individual predictions. In all figures, "MA" refers to the moving average, whilst "Delta" refers to the percentage change in the value from the previous day. Additionally, all figures show the breakdown of single predictions and how each feature contributed to that prediction based on SHAP values. Here, the values on the arrows correspond to the normalised feature value in units of standard deviations away from the mean. These were calculated on the "Date-ID Split" test set.

Table 3: Mean (95% CI) % of the accuracy on the Male and Female participants on the test set of the different data splits with 10 bootstrap repeats. We also show the ratio of positive and negative labels in each demographic and the likelihood of a positive model prediction.

		Accuracy	No. of Participants	Positive : Negative	$\Pr(\hat{Y} = 1 \mid \text{Sex})$
Date	Female	52.6 (31.8 - 73.4)	16	1 : 1.7	35.5 (32.4 - 38.5)
	Male	86.5 (76.2 - 96.9)	25	1 : 5.5	32.3 (30.9 - 33.8)
Date-ID	Female	54.6 (26.8 - 82.4)	11	1 : 2.1	37.4 (33.6 - 41.1)
	Male	85.3 (72.9 - 97.7)	20	1 : 4.1	38.0 (36.4 - 39.7)

[6] Breiman L. Random Forests. Machine Learning. 2001;45(1):5-32. Available from: `https://doi.org/10.1023/a:1010933404324`.

[7] Pedregosa F, Varoquaux G, Gramfort A, Michel V, Thirion B, Grisel O, et al. Scikit-learn: Machine Learning in Python. Journal of Machine Learning Research. 2011;12:2825-30.

[8] Paszke A, Gross S, Massa F, Lerer A, Bradbury J, Chanan G, et al. PyTorch: An Imperative Style, High-Performance Deep Learning Library. In: Advances in Neural Information Processing Systems 32. Curran Associates, Inc.; 2019. p. 8024-35.

[9] DeGroot MH, Fienberg SE. The Comparison and Evaluation of Forecasters. The Statistician. 1983 Mar;32(1/2):12.

[10] Niculescu-Mizil A, Caruana R. Predicting good probabilities with supervised learning. In: Proceedings of the 22nd international conference on Machine learning - ICML '05. Bonn, Germany: ACM Press; 2005. p. 625–632. Available from: `http://portal.acm.org/citation.cfm?doid=1102351.1102430`.

[11] Guo C, Pleiss G, Sun Y, Weinberger KQ. On Calibration of Modern Neural Networks. In: Precup D, Teh YW, editors. Proceedings of the 34th International Conference on Machine Learning. vol. 70 of Proceedings of Machine Learning Research. PMLR; 2017. p. 1321-30. Available from: `https://proceedings.mlr.press/v70/guo17a.html`.

[12] Anger J, Lee U, Ackerman AL, Chou R, Chughtai B, Clemens JQ, et al. Recurrent Uncomplicated Urinary Tract Infections in Women: AUA/CUA/SUFU Guideline. Journal of Urology. 2019 Aug;202(2):282–289.

[13] Deltourbe L, Lacerda Mariano L, Hreha TN, Hunstad DA, Ingersoll MA. The impact of biological sex on diseases of the urinary tract. Mucosal Immunology. 2022 Aug;15(5):857–866.

[14] Mehrabi N, Morstatter F, Saxena N, Lerman K, Galstyan A. A Survey on Bias and Fairness in Machine Learning. ACM Computing Surveys. 2022 Jul;54(6):1–35.

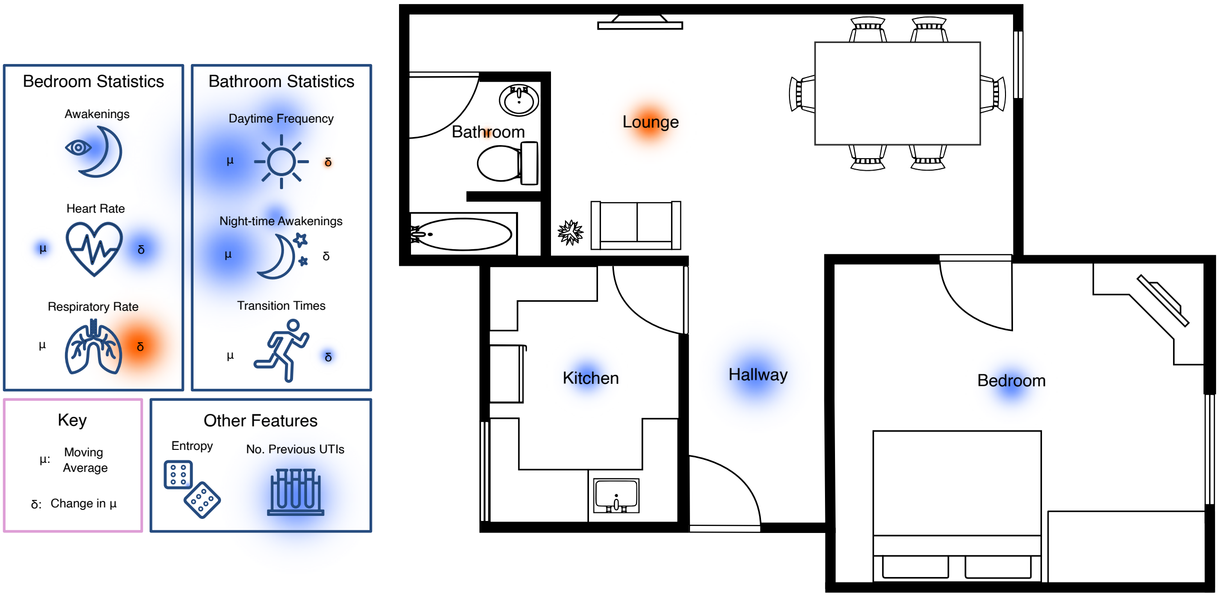

Supplementary Figure 10: SHAP values projected on the house layout for a single correct positive prediction. The intensity of the colours shows the relevance of the sensory values to the UTI risk scores (orange representing positive correlations and blue representing negative correlations).

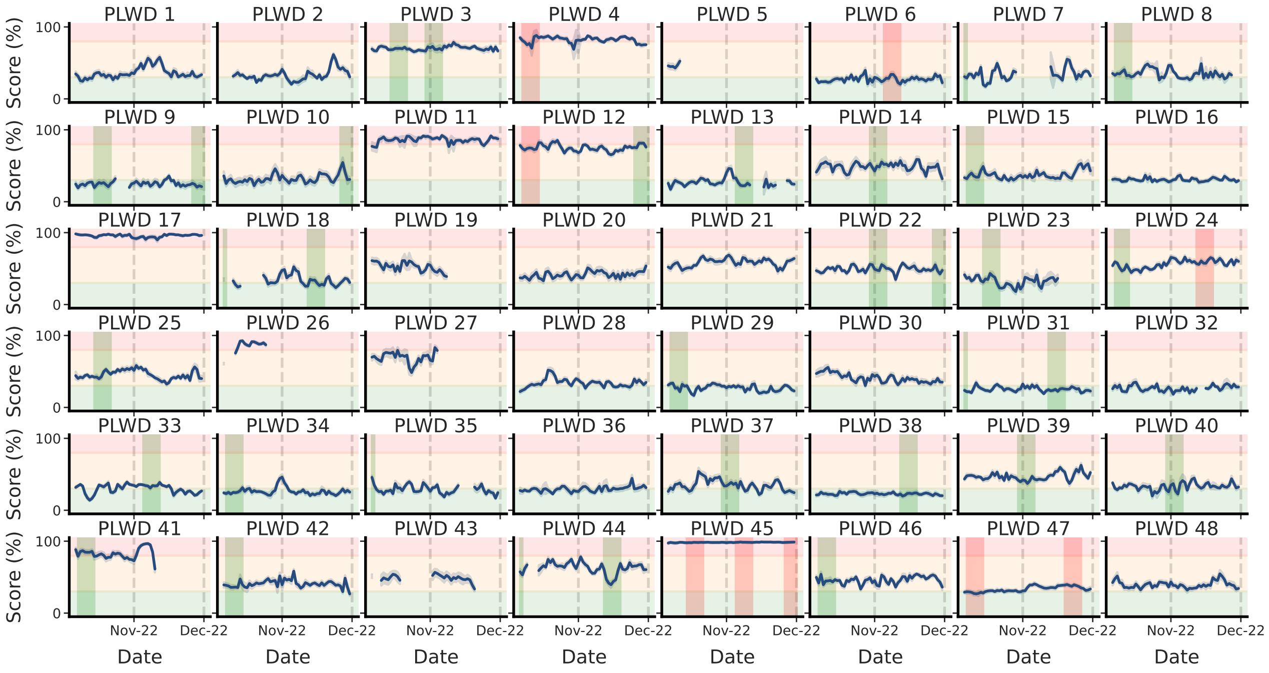

Supplementary Figure 11: UTI risk scores on date-ID split test set. The risk scores as calculated by the best model on the test set of the "Date-ID Split", with the thresholds corresponding to the horizontal coloured bands and the vertical coloured bands representing positive and negative UTI diagnoses (in Red and Green respectively). The error bands represent the 95% confidence interval (1000 bootstrap samples) of the mean of the values from the 10 bootstrap repeats.

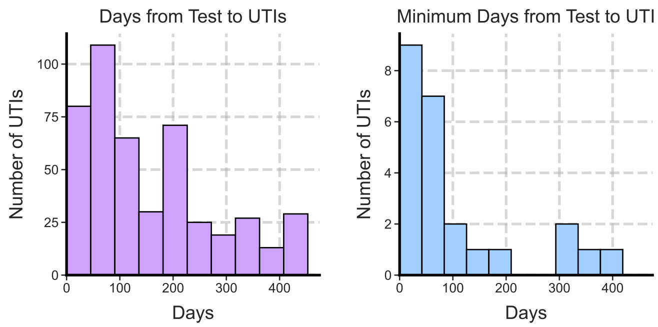

(a) (b)

Supplementary Figure 12: Characteristics of UTIs in the test set. a Histogram showing the positively labelled UTIs and the number of days they were measured before the start of the test set. **b** The same data as **a**, presented as a minimum number of days for each participant.

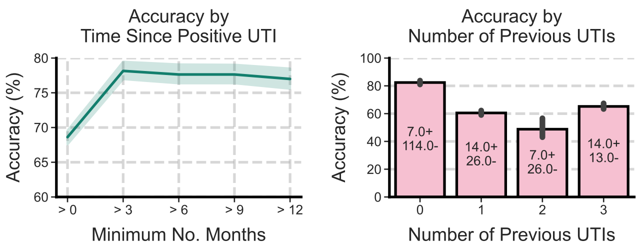

(a) (b)

Supplementary Figure 13: Accuracy by time since, and number of UTIs. a The accuracy of the predictions on participants (on the test set of “Date Split”) that had a positive UTI label at least the given number of months earlier than the test set. The result for those > 12 months also contains predictions on those participants that did not have a positive label prior to the test set. The error bands represent the 95% confidence interval (1000 bootstrap samples) of the mean of the values from the 10 bootstrap repeats. **b** The accuracy (on the test set of “Date-ID Split”) split by the number of previous UTIs of the participants had. The labels on the bars represent the number of positively and negatively labelled data points (in the test set) for the corresponding group. The error bars represent the 95% confidence interval (1000 bootstrap samples) of the mean of the values from the 10 bootstrap repeats.
