# Supplementary material for: Digital remote monitoring for screening and early detection of urinary tract infections
Source: NPJ Digit Med. 2024 Jan 13;7:11. doi: 10.1038/s41746-023-00995-5 (PMC10787784; doi:10.1038/s41746-023-00995-5)
Supplement: Supplementary file 3 — Reporting Summary [file 41746_2023_995_MOESM3_ESM.pdf]

nature portfolio

Corresponding author(s): Alexander Capstick
Last updated by author(s): Nov 22, 2023

Reporting Summary

Nature Portfolio wishes to improve the reproducibility of the work that we publish. This form provides structure for consistency and transparency in reporting. For further information on Nature Portfolio policies, see our Editorial Policies and the Editorial Policy Checklist.

Statistics

For all statistical analyses, confirm that the following items are present in the figure legend, table legend, main text, or Methods section.

n/a	Confirmed	
☐	☒	The exact sample size (*n*) for each experimental group/condition, given as a discrete number and unit of measurement
☒	☐	A statement on whether measurements were taken from distinct samples or whether the same sample was measured repeatedly
☒	☐	The statistical test(s) used AND whether they are one- or two-sided<br>*Only common tests should be described solely by name; describe more complex techniques in the Methods section.*
☐	☒	A description of all covariates tested
☐	☒	A description of any assumptions or corrections, such as tests of normality and adjustment for multiple comparisons
☐	☒	A full description of the statistical parameters including central tendency (e.g. means) or other basic estimates (e.g. regression coefficient) AND variation (e.g. standard deviation) or associated estimates of uncertainty (e.g. confidence intervals)
☒	☐	For null hypothesis testing, the test statistic (e.g. *F*, *t*, *r*) with confidence intervals, effect sizes, degrees of freedom and *P* value noted<br>*Give P values as exact values whenever suitable.*
☒	☐	For Bayesian analysis, information on the choice of priors and Markov chain Monte Carlo settings
☒	☐	For hierarchical and complex designs, identification of the appropriate level for tests and full reporting of outcomes
☒	☐	Estimates of effect sizes (e.g. Cohen's *d*, Pearson's *r*), indicating how they were calculated

Our web collection on statistics for biologists contains articles on many of the points above.

Software and code

Policy information about availability of computer code

Data collection	Data was obtained from patients using a system developed by the UK Dementia Research Institute called Minder. A data snapshot was then extracted using DCARTE (https://github.com/esoreq/dcarte/), which is an open-source python package developed by Dr Eyal Soreq at the UK Dementia Research Institute.
Data analysis	Data analysis was performed using python 3.9 installed within an anaconda environment, and with the packages: numpy, pandas, scikit-learn, matplotlib, seaborn, pytorch. On request we will supply a copy of the anaconda environment file and code.

For manuscripts utilizing custom algorithms or software that are central to the research but not yet described in published literature, software must be made available to editors and reviewers. We strongly encourage code deposition in a community repository (e.g. GitHub). See the Nature Portfolio guidelines for submitting code & software for further information.

Data

Policy information about availability of data

All manuscripts must include a data availability statement. This statement should provide the following information, where applicable:

- Accession codes, unique identifiers, or web links for publicly available datasets
- A description of any restrictions on data availability
- For clinical datasets or third party data, please ensure that the statement adheres to our policy

The data that support the findings of this study are available from the corresponding author upon reasonable request.

Research involving human participants, their data, or biological material

Policy information about studies with human participants or human data. See also policy information about sex, gender (identity/presentation), and sexual orientation and race, ethnicity and racism.

Reporting on sex and gender	Sex was reported within the cohort demographics table in the manuscript and model performance was analysed by sex so that model fairness could be measured.
Reporting on race, ethnicity, or other socially relevant groupings	Ethnicity was reported in the cohort demographics table in the manuscript. These were self-reported.
Population characteristics	Information about age, whether the participant lives alone, and their dementia diagnosis were collected as part of the study. These are reported within the cohort demographics.
Recruitment	Participants are recruited via NHS services. Capacity of both the participant and study partner is assessed at each research visit. Research staff conducting the assessment have completed the NIHR GCP training and Valid Informed Consent training. If a participant is deemed to lack capacity but is willing to take part in the research, a personal consultee is sought in the first instance to sign a declaration of consent. If no personal consultee can be found, a professional consultee, such as a key worker, is sought. This process is included in the study protocol and ethical panel approval is obtained.
Ethics oversight	The study's oversight and governance is conducted by the Surrey and Borders NHS Trust and led by Prof Ramin Nilforooshan. The study protocol for this work was reviewed and approved by an independent NHS Ethics Committee and given Health Research Authority approval. The Integrated Research Application System (IRAS) ID for the study is: 257561.

Note that full information on the approval of the study protocol must also be provided in the manuscript.

Field-specific reporting

Please select the one below that is the best fit for your research. If you are not sure, read the appropriate sections before making your selection.

☒ Life sciences ☐ Behavioural & social sciences ☐ Ecological, evolutionary & environmental sciences

For a reference copy of the document with all sections, see nature.com/documents/nr-reporting-summary-flat.pdf

Life sciences study design

All studies must disclose on these points even when the disclosure is negative.

Sample size	Sample size was determined by the number of people recruited by the Minder study who had a positive or negative UTI diagnosis between June 2020 and December 2022. No sample-size calculation was performed. We collected enough data and labels to train simple machine learning models and perform proof-of-concept analysis. This is because we have 20 features and 1752 examples.
Data exclusions	Exclusion criteria was pre-established for our study and were as follows: (1) patients receiving treatment for terminal illness (2) presence of severe mental health conditions including depression, anxiety, psychosis, and agitation (3) presence of active suicidal thoughts.
Replication	Data was split into training, validation, and testing sets from based on dates to ensure that there was no data leakage. Models were then trained 10 times (with bootstrap sampling) on the training data and the validation set was used to tune model parameters. Models were then trained again on the training and validation set together 10 times (with bootstrap sampling) and then each model was evaluated on the test set. This ensures that the results are resilient to changes in the training data. We evaluated the models on the test set in two different ways. The first involved the above procedure and the second involved a leave-one-out training and testing routine, in which the model was trained on all participants except that seen at test time, and this was repeated for all participants (with 10 bootstrap runs per participant). This ensured that the model still performed well when it was predicting on participants that it has not seen historical data for.
Randomization	Splitting the data points into training and testing sets randomly might have led to shortcuts in the model and cause the model to learn how to separate participants rather than labels. Additionally, splitting participants randomly into training and testing sets is unrealistic when considering how the model might work in practice, since the dates of the examples collected will be mixed within the training and testing sets, leading to non-generalisable results. We therefore opted to split the data by date, since this is how the model would be used in practice. We then performed two evaluation methods to test the model's ability to learn and predict labels from participants that it has seen historical data for and perform a leave-one-out evaluation (over all participants in the test set) to understand the performance of the model when predicting on new participants.
Blinding	All data analysed was anonymised and population characteristics were only used for analysis of results and not during model training or development.

Reporting for specific materials, systems and methods

We require information from authors about some types of materials, experimental systems and methods used in many studies. Here, indicate whether each material, system or method listed is relevant to your study. If you are not sure if a list item applies to your research, read the appropriate section before selecting a response.

Materials & experimental systems

n/a	Involved in the study
☒	☐ Antibodies
☒	☐ Eukaryotic cell lines
☒	☐ Palaeontology and archaeology
☒	☐ Animals and other organisms
☒	☐ Clinical data
☒	☐ Dual use research of concern
☒	☐ Plants

Methods

n/a	Involved in the study
☒	☐ ChIP-seq
☒	☐ Flow cytometry
☒	☐ MRI-based neuroimaging
